# Effects of a Parent-Child Single-Session Growth Mindset Intervention on Adolescent Depression and Anxiety Symptoms: Protocol of a 3-Arm Waitlist Randomized Controlled Trial

**DOI:** 10.2196/63220

**Published:** 2024-08-30

**Authors:** Shimin Zhu, Yuxi Hu, Ruobing Wang, Di Qi, Paul Lee, So Wa Ngai, Qijin Cheng, Paul Wai Ching Wong

**Affiliations:** 1 Department of Applied Social Sciences The Hong Kong Polytechnic University Hong Kong China (Hong Kong); 2 Mental Health Research Centre The Hong Kong Polytechnic University Hong Kong China (Hong Kong); 3 Southampton Clinical Trials Unit University of Southampton Southampton United Kingdom; 4 Department of Social Work The Chinese University of Hong Kong Hong Kong China (Hong Kong); 5 Department of Social Work and Social Administration The University of Hong Kong Hong Kong China (Hong Kong)

**Keywords:** implicit theory, fixed mindset, mental health, secondary school students, belief in change

## Abstract

**Background:**

Depression and anxiety are common mental health problems among adolescents worldwide. Extant research has found that intelligence, emotion, and failure-is-debilitating beliefs (fixed mindsets) are closely related to more depression and anxiety symptoms, hopelessness, and suicidality. Recent research also points to the importance of parental mindset, which can strongly influence children’s affect, behavior, and mental health. However, the effects of parent-child mindset interventions on a child’s internalizing problems have not yet been empirically examined. As recent evidence has shown the promise of single-session interventions in reducing and preventing youth internalizing problems, this study develops and examines a parent and child single-session intervention on mindsets of intelligence, failure, and emotion (PC-SMILE) to tackle depression and anxiety in young people.

**Objective:**

Using a 3-arm randomized controlled trial, this study will examine the effectiveness of PC-SMILE in reducing depression and anxiety symptoms among children. We hypothesize that compared to the waitlist control group, the PC-SMILE group and child single-session intervention on mindsets of intelligence, failure, and emotion (C-SMILE) group will significantly improve child depression and anxiety (primary outcome) and significantly improve secondary outcomes, including children’s academic self-efficacy, hopelessness, psychological well-being, and parent-child interactions and relationships, and the PC-SMILE is more effective than the C-SMILE.

**Methods:**

A total of 549 parent-child dyads will be recruited from 8 secondary schools and randomly assigned to either the PC-SMILE intervention group, the C-SMILE intervention group, or the no-intervention waitlist control group. The 45-minute interventions include parent-version and child-version. Both parents and students in the PC-SMILE group receive the intervention. Students in C-SMILE group receive intervention and their parents will receive intervention after all follow-up ends. Students in 3 groups will be assessed at 3 time points, baseline before intervention, 2 weeks post intervention, and 3 months post intervention, and parents will be assessed in baseline and 3-month follow-up. The intention-to-treat principle and linear-regression-based maximum likelihood multilevel models will be used for data analysis.

**Results:**

Recruitment started in September 2023. The first cohort of data collection is expected to begin in May 2024 and the second cohort will begin in September 2024. The final wave of data is expected to be collected by the end of the first quarter of 2025. The results are expected to demonstrate improved anxiety and depression among students assigned to the intervention condition, as well as the secondary outcomes compared to those in the control group. The efficacy and effectiveness of the intervention will be discussed.

**Conclusions:**

This study is the first attempt to develop a web-based single-session intervention for students and their parents to enhance their well-being in Hong Kong and beyond, which potentially contributes to providing evidence-based recommendations for the implementation of brief digital parent-child interventions.

**International Registered Report Identifier (IRRID):**

PRR1-10.2196/63220

## Introduction

### Background

Depression and anxiety are the leading causes of disability among adolescents worldwide [1]. In Hong Kong, 1 in 4 secondary school students report moderate to severe depression or anxiety symptoms [2]. Around 1 in 12 have been diagnosed with severe depression or anxiety disorders [3]. It is estimated that about 43,500 to 87,000 secondary students need support or intervention to manage their symptoms [4], but 65% of these students fail to access mental health services due to lengthy waitlists, costly clinic-based treatments, or concerns of stigmatization or discrimination [5]. Developing these disorders early in life can increase young people’s suicidal risk and lead to a wide range of psychological and vocational impairments that result in harmful long-term effects [6], especially if left untreated or given insufficient support. Thus, there is a need for integrated and effective interventions that are acceptable to families, cost-effective, and nonstigmatizing to tackle these disorders by providing early intervention and preventing deterioration.

There is substantial, robust evidence on risk and protective parenting factors for adolescent anxiety and depression [7,8]. Beyond structural elements such as academic stress [7], increasing evidence highlights the importance of parent and child belief systems, which are potentially modifiable. Mindset studies have identified that a fixed mindset (ie, believing one’s attributes are unchangeable) is a key predictor of youth mental health symptoms [9-12], while interventions promoting a growth mindset (ie, believing one’s attributes are changeable) effectively reduce adolescent depression and anxiety symptoms [13,14]. Research has also revealed that parents’ fixed mindset regarding intelligence and emotions can lead to more internalizing problems such as depression and anxiety among children and lower perceived control over mental health symptoms [15-18]. Children in fixed-mindset households might question their abilities, use ineffective coping strategies, and experience greater helplessness [9].

Research on implicit theory has found that people who believe that personal attributes can be developed (also referred to as growth mindset) are more likely to thrive in the face of difficulty and continue to improve. In contrast, those who hold a more fixed mindset may shy away from challenges or fail to meet their potential [19,20]. A growth mindset intervention’s core content teaches that people’s attributes, such as intelligence, personality, or emotions, can change and that people can improve through the actions they take (eg, making an effort, changing strategies, learning from failures, and asking for help) [19,21,22]. Emphasizing the potential to change in mindset interventions of 30 minutes to 1 hour resulted in different change effect sizes, from *d*=0.1 in changing school performance in a large-scale national study among heterogenous participants [23] to *d*=0.59 in randomized controlled trials (RCTs) on reducing anxiety [14]. Considering the brevity of the interventions and the effect sizes in relation to heterogeneity and contextual factors, low-intensity interventions on mindset have demonstrated high potential scalability for mental health prevention and early intervention measures.

Well-crafted growth mindset interventions promoting a core growth mindset are crucial to securing the effects of the intervention [19]. Existing interventions have focused on growth mindsets for intelligence [19,23], personality [14,24], and emotion [22] to promote school performance and resilience and to reduce mental health symptoms. Although these domain-specific growth mindset interventions have achieved positive improvements, there has never been an integrated intervention addressing multiple core growth mindsets. The factors for youth depression and anxiety are multifaceted, such as academic stress, failures and uncertainty in life, and the emotional struggles of puberty. Teaching multiple relevant growth mindsets in 1 intervention may address several key risk factors and strengthen the potency of growth mindset interventions to better reduce youth depression and anxiety symptoms. The proposed study will address this research gap by developing and examining an integrated intervention targeting growth mindsets for youth internalizing symptoms.

Parental mindsets matter for adolescents’ mindsets and internalizing problems such as depression and anxiety [16,17]. Parents are the most direct social support for adolescents, but parents can also be a source of stress if they hold a fixed mindset and think failure is debilitating. For example, parental fixed mindsets about intelligence predict depression symptoms and social anxiety as children have more performance worries and are afraid of negative evaluation [17]. Parents’ failure mindset also influences parenting practices in the face of setbacks [16]. A fixed emotion mindset and failure-is-debilitating beliefs in parents were associated with passive help-seeking preferences for children’s mental health symptoms [18]. In contrast, building belief-in-change about intelligence and emotion may relieve family concerns about failure, mental health symptoms, and stigmatization, increase perceived control and active help-seeking, and build better parent-child relationships. As parents play a crucial role in preventing children’s internalizing problems [25], joint parent-child growth mindset intervention is warranted and promising for promoting both parental and child well-being.

Although there is evidence of the importance of parents and children’s mindsets in internalizing symptoms, we have identified 3 important research gaps which will be tackled in the present study. First, current interventions usually focus on fixed mindsets of a single domain—such as intelligence, personality, or emotion [14,22]—but have rarely used a few relevant mindset domains in one intervention, which are closely related to youth mental health. An integrated mindset intervention is very likely to strengthen intervention efficacy, especially for brief interventions. Second, although there is an ongoing parent-child mindset intervention to promote treatment access in the United States [26], there is no such intervention in the Chinese context. In Chinese culture, parents are very anxious about their children’s education [27], and parental mindset is more likely to influence children’s mental health [28]. Third, although evidence highlights the remarkable promise of preventive parenting interventions, parenting programs are often not used even when available because of barriers such as scheduling difficulties and privacy concerns [6]. Thus, it is important to develop brief, accessible, and nonstigmatizing interventions for parents and children.

The current study will therefore develop and examine a web-based parent and child single-session intervention on mindset of intelligence, failure, and emotion (PC-SMILE). The research question is if the PC-SMILE effectively reduce depression and anxiety symptoms among secondary school students and how does it impact academic self-efficacy, psychological well-being, hopelessness, and parent-child interactions and relationships.

#### Objectives

The primary objective of this study is to evaluate the effectiveness of the PC-SMILE in reducing depression and anxiety symptoms in secondary school students (primary outcome). The secondary objective is to examine the effectiveness of the PC-SMILE in (1) enhancing academic self-efficacy, enhancing psychological well-being, and reducing hopelessness; and (2) enhancing parent-child interactions and relationships (secondary outcomes).

#### Hypotheses

This study will examine the effectiveness of the PC-SMILE for adolescents and their parents by comparing it to a child single-session intervention on mindset of intelligence, failure, and emotion (C-SMILE) intervention group that provides the child-version intervention to children only and a no-intervention waitlist control group.

Our hypotheses are the following:

H1: The PC-SMILE and C-SMILE groups have better improved primary outcome compared to the waitlist control group, including reducing anxiety and depression symptoms in student participants, while PC-SMILE is more effective than C-SMILE.

H2: The PC-SMILE and C-SMILE are more effective in the secondary outcomes of (1) reducing hopelessness, (2) enhancing academic self-efficacy, (3) enhancing psychological well-being, and (4) enhancing parent-child interactions and relationships than waitlist control, and PC-SMILE is more effective than C-SMILE.

## Methods

### PC-SMILE Intervention

Although single-session interventions (SSIs) are a relatively new intervention approach for youth mental health, rich empirical evidence has suggested the promise of SSIs for youth mental health promotion in matters such as increasing hope and happiness [29]; adaptive coping with peer stressors [30]; and improving perceived control, stress recovery, and anxiety and depression symptoms [14]. According to the literature on promoting SSI effectiveness [19,31,32] and our experience in developing SSIs in the Chinese culture [22], the PC-SMILE was designed with 5 important features to ensure efficacy.

First, the PC-SMILE addresses core beliefs related to youth depression and anxiety. The selection of intelligence, emotion, and failure mindset is based on the extant literature [17,33-36] and our fieldwork in schools based on parent and public involvement. By instilling the belief that “intelligence and negative emotions are changeable” and “failure is necessary for personal growth,” this innovative integrated intervention has a high potential to relieve parents and children’s stress and to promote well-being.

Second, the PC-SMILE was designed based on concepts and findings based on neuroscience to normalize the concepts of a growth mindset; for example, the concept of neuroplasticity is introduced to explain how and why personal traits are malleable. The core message is conveyed to participants through scientific videos and memorable metaphors. The PC-SMILE uses the muscle metaphor (ie, the brain is like a muscle, it becomes stronger and smarter when you exercise it) [19] for both students and parents, and it uses the planting metaphor for parents to emphasize the importance of patience, respect for individual differences and support for children. Illustrating ideas with metaphors is more likely to motivate sustained behavioral change [19].

Third, PC-SMILE empowers both students and their parents to act as “helpers” or “experts” by framing the participants as active contributors to the improvement of the intervention and by asking them to provide support to peers [14,32]. This design aligns with adolescents’ desire for respect [31].

Fourth, as in previous interventions [14,32], storytelling is a powerful feature of the PC-SMILE. Participants learn the stories from valued others, such as older peers and experts, which is helpful in conveying the core messages of the growth mindset. We also use the “saying-is-believing” exercise to invite the participants to share their learnings from the intervention with future intervention users. This exercise helps participants to internalize the growth mindset through cognitive dissonance processes [19,37].

Fifth, as the effects of SSIs may wane over time, booster messages will be adopted in the PC-SMILE. Weekly booster reminders [38] of core growth mindset quotations will be sent to both parent and child participants via email or text messages to sustain the mindset and behavioral changes.

### Research Design

**Figure 1:**
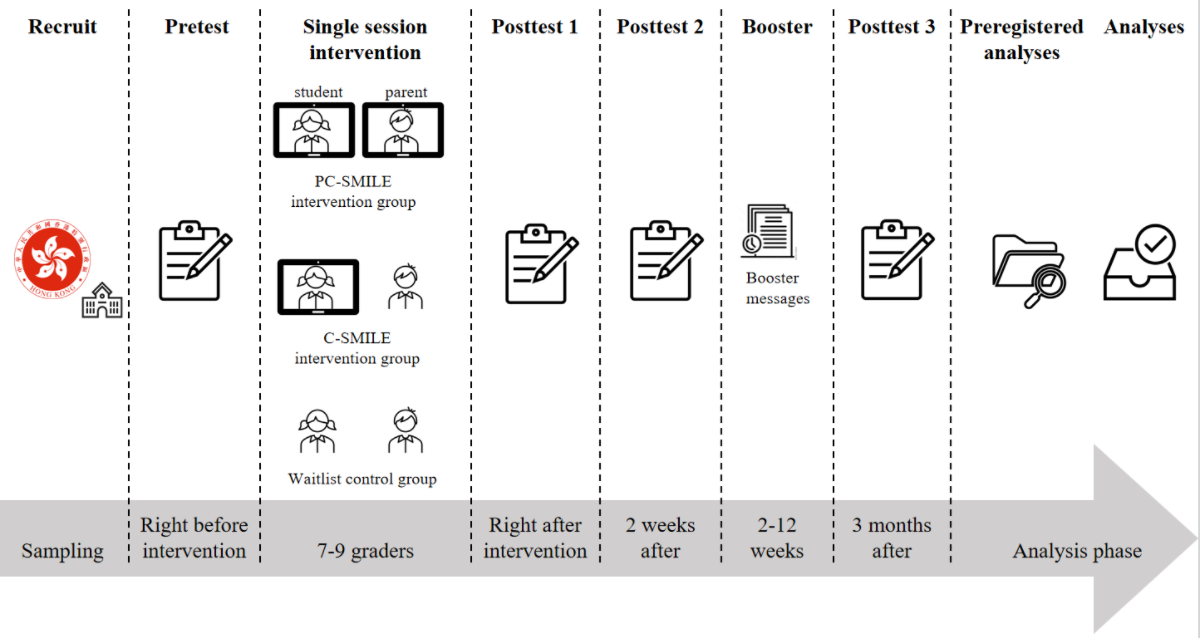
Design of the 3-arm waitlist randomized controlled trial. C-SMILE: child single-session intervention on mindset of intelligence, failure, and emotion; PC-SMILE: parent and child single-session intervention on mindset of intelligence, failure, and emotion.

**Figure 2:**
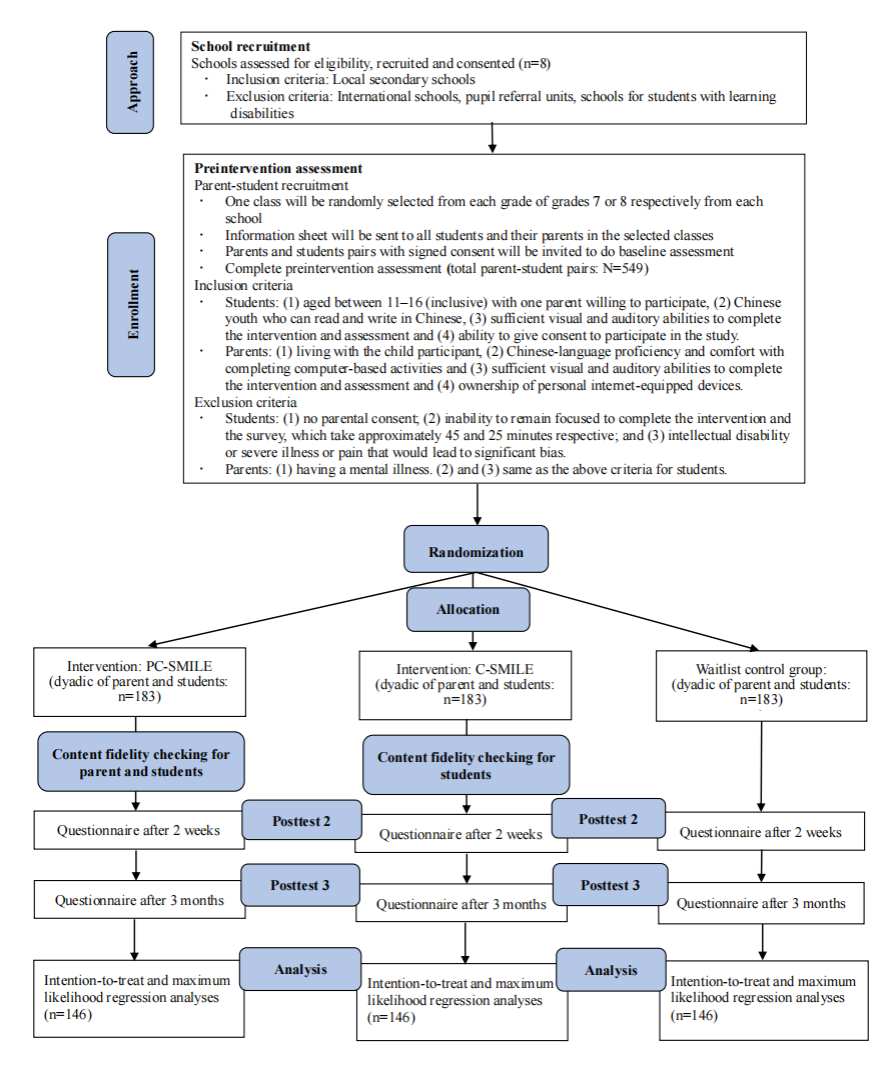
Consolidated Standards of Reporting Trials (CONSORT) flow diagram. C-SMILE: child single-session intervention on mindset of intelligence, failure, and emotion; PC-SMILE: parent and child single-session intervention on mindset of intelligence, failure, and emotion.

This study will be a 3-arm cluster-RCT with a waitlist control group design. A conceptual model is provided in Figure 1. The intervention protocol will strictly follow the CONSORT (Consolidated Standards of Reporting Trials) guidelines [39,40] and has been preregistered before data collection in Open Science Framework and ClinicalTrials.gov. Classes in each eligible school will be randomized (using computer-generated random numbers) into the PC-SMILE intervention, C-SMILE, or waitlist group. The waitlist group will undergo intervention after the 3-month postintervention survey. All student participants will receive regular educational activities and interventions in school. A total of 3 repeated assessments of the measures will be conducted simultaneously for the children in three groups at (1) baseline, (2) 2-weeks post intervention, and (3) 3-months post intervention (Multimedia Appendix 1). The CONSORT flow diagram is provided in Figure 2. Cluster randomization will be conducted at the class level.

### Sample Size Determination

#### Overview

We assume a small to medium effect size (Cohen *d*=0.33) of the current intervention according to a meta-analysis of SSIs addressing youth psychiatric problems [13], a power of 0.80 and an α of .05. A sample size of 438 (146 per arm) is required to detect statistical significance. With reference to the approximately 20% attrition rate of our previous studies in the school setting, 549 pairs of parent-student participants will be recruited at the baseline (183 per arm). Our existing research suggests that this is feasible within the timeframe.

#### Participants and the Randomized Control Process

Eligible adolescents in grades 7-8 and their parents will be recruited from 8 secondary schools through cluster-randomized sampling. The inclusion criteria for adolescent participants will include (1) aged between 11-16 years (inclusive) with 1 parent willing to participate; (2) Chinese youths who can read and write in Chinese language; (3) sufficient visual and auditory abilities to complete the intervention and assessment; and (4) ability to give consent to participate in the study. Exclusion criteria for adolescent participants will include (1) no parental consent; (2) inability to remain focused to complete the intervention and the survey, which take approximately 45 and 25 minutes respectively; and (3) intellectual disability or severe illness or pain that would lead to significant bias in students’ health and mental health situation.

Inclusion criteria for parent participants are (1) living with the child participant; (2) Chinese-language proficiency and comfort with completing computer-based activities; (3) sufficient visual and auditory abilities to complete the intervention and assessment; and (4) ownership of personal internet-equipped devices. Exclusion criteria for parent participants include (1) having a mental illness as well as exclusion criteria (2) and (3) from the aforementioned criteria for adolescent participants.

The school and student recruitment process will include the following steps. First, we will send research invitations to schools chosen randomly from the secondary school list covering 3 areas of Hong Kong (ie, Hong Kong Island, Kowloon, and the New Territory). Invitations will not stop until 8 schools agree to participate. Then, 3 classes from grades 7 or 8 will be selected by a random number, and all students in the selected class will be invited to join the study. There are generally 35 students in 1 class. We estimate that approximately 25 pairs of parents and students will join the study. If too few participants join the study, the random class selection process will be repeated until 70 pairs of eligible parent-child participants in each school are recruited. Using random numbers, the selected 3 classes will be assigned to the PC-SMILE intervention group, the C-SMILE intervention group, and the waitlist control group. If the targeted sample size is met, the school invitation and recruitment will stop.

Students and parents will complete all study procedures via personal internet-equipped devices. Parent-student pairs will be assigned to the same condition (the PC-SMILE or C-SMILE or waitlist). They will receive the link of research material and complete Qualtrics-based baseline survey, intervention, and follow-up surveys independently. For student participants, the interventions will be conducted in school classrooms or activity rooms that offer sufficient computers or tablets, and headphones. All intervention activities will be self-administered by youths and delivered in a web-based format. The research team and trained research assistants will remain in the intervention rooms to provide help if needed. Parent participants will receive a study link with the baseline Qualtrics survey and the intervention. The interventions for parents and students are different in terms of narrative and content. Between the 2-week posttest and the 3-month follow-up survey, a total of 5 weekly booster messages with core intervention content will be sent to the intervention groups every 2 weeks. After completion of the 3-month follow-up, parents in the C-SMILE or the control group who do not initially receive the intervention will be granted the intervention access.

### Intervention Groups

#### PC-SMILE Group

The PC-SMILE integrates the growth mindsets of intelligence, failure, and negative emotions and introduces these growth mindsets to students and parents. The interventions for both students and parents consist of five components: (1) an introduction to brain functions regarding the potential of neuroplasticity and the possibility of changes in intelligence and emotions, (2) stories and testimonials from school-aged youths who describe their belief-in-change, (3) short videos with stories of improving intelligence and emotions and of failure-is-enhancing, (4) common questions and misconceptions about growth mindset, and (5) self-persuasion writing exercises in which the participants write notes to young students or others about the growth mindsets. The interventions for parents and students are different in terms of narrative and content. Between the 2-week posttest and the 3-month follow-up survey, a total of 5 weekly booster messages with core intervention content will be sent to the intervention groups every 2 weeks. The interventions for children and parents last 45 minutes.

#### C-SMILE Group

In the C-SMILE group, only students receive the child-version intervention, but their parents do not receive parent-version intervention until the 3-month follow-up is finished. Parents are invited to do the preintervention survey and the 3-month follow-up survey.

### Waitlist Control Group

The waitlist control group will continue with normal education activities and do the preintervention and follow-up surveys at the same timeframe as the intervention groups. Participants and their parents in the waitlist group will be invited to complete the PC-SMILE after the 3-month follow-up survey.

### Measurements

Participants will be invited to read the instructions of the survey and complete self-rated scales. All the measures have been pilot-tested, and the questionnaires take approximately 25 minutes to complete.

Mindset about intelligence will be measured using the 3-item Implicit Theory of Intelligence Scale, which measures beliefs about the malleability of intelligence (eg, “You have a certain amount of intelligence and there is really not much you can do to change it”) [41,42]. Each item is rated on a 6-point scale (1=strongly disagree to 6=strongly agree). A higher score represents a more fixed mindset. The Cronbach α in the previous study was 0.87 with good validity [42,43].

Mindset about failure will be measured by the 6-item Implicit Theory of Failure Scale [16], which measures views of failure as enhancing versus debilitating on a 6-point Likert scale (1=strongly disagree to 6=strongly agree). A sample item of an enhancing belief is “The effects of failure are positive and should be used,” and a sample item of a debilitating belief is “The effects of failure are negative and should be avoided*.*” Enhancing belief items will be reverse scored. A higher score reflects more debilitating beliefs. The Cronbach α of the parent survey in the previous study was 0.88 [16].

Mindset about emotion will be assessed using the validated Chinese version of the 12-item Mindset of Depression, Anxiety, and Stress Scale [35] to measure children’s belief in the change of negative emotion states. Sample items include “When you have a certain level of depression/anxiety/stress, you really cannot do much to change it.” Parents’ mindset about emotion will be measured by a 4-item version of Mindset of Depression, Anxiety, and Stress Scale, which includes sample item “If they want to, people can change the emotions they have.” Each item is scored on a 6-point Likert scale (1=strongly disagree to 6=strongly agree), and a higher score means a more fixed mindset (Cronbach α=0.94) [35].

### Primary Outcome

Children’s depression and anxiety symptoms will be measured using the validated 25-item Revised Children’s Anxiety and Depression Scale for both children and parents [44,45]. Sample items measuring anxiety and depression include “I/My child worry when I/My child think I/My child have done poorly at something” and “‘I/My child feel sad or empty.” The frequency of each item is rated on a 4-point Likert scale with reference to the past 2 weeks (0=never to 3=always). The child survey has an overall score, total anxiety score from the anxiety subscale (Cronbach α=0.94), and total depression score from the depression subscale (Cronbach α=0.79) [44].

### Secondary Outcomes

The 4-item Hopelessness Scale [46,47] will be used to measure children and parents’ faith in the future. Each item is scored on a 5-point Likert scale from 1=strongly disagree to 5=strongly agree, and the mean of all 4 items is taken to measure hopelessness, with a higher score corresponding to a higher level of hopelessness. An example item is “My future seems dark to me.” The Cronbach α was 0.834 with good validity [48].

The validated Chinese version of Short Warwick-Edinburgh Mental Well-Being Scale [49,50] will be used to measure children’s and parents’ general experience of well-being. The Short Warwick-Edinburgh Mental Well-Being Scale includes 7 items scored on a 5-point Likert scale from 1=none of the time to 5=all the time, and the average of all 7 items indicates participants’ well-being. A higher averaged score represents a higher level of well-being. A sample item is “I have been feeling optimistic about the future.” The Cronbach α was 0.93 [50].

Parent-child interactions will be assessed by 3 self-developed questions about how many days the children spend more than 15 minutes per day doing activities with their parents in a week. Children will answer the frequency by the number of days from 0=none to 7=seven days. Parenting activities include chatting, handing out, and watching television or movies or videos. Student participants who rate higher frequency of the parenting activities have better interactions with parents. Parent-child relationships will be measured using 3 self-developed items. Questions are “I am very satisfied with the relationship between me and my parents,” “I proactively tell my family what had happened to me,” and “I proactively share my feelings with my family” for adolescents. The adapted items “My children are very satisfied with the relationship between them and their parents,” “My children proactively tell me what had happened to them,” and “My children proactively share their feelings with me” are for parents. Each item is rated on a 4-point Likert scale (1=strongly disagree to 4=strongly agree). A higher score indicates a better parent-child relationship.

Perceived parent learning versus performance orientation will be assessed from both children and parents’ perspectives. Child-version has 8 items and parent-version has 12 items, with half of the items measuring performance-orientation and half measuring learning orientation mindset, respectively [16]. The scale asks participants about the parents’ responses to a scenario when their child gets a poor grade on a quiz. Sample items of performance-orientation are “My parents don’t like it when I make mistakes in school” (child-version) and “I might worry (at least for a moment) that my child isn't good at this subject” (parent-version). Sample items of learning-orientation are “My parents want me to understand school concepts, not just do the work” (child-version) and “I’d discuss with my child whether it would be useful to ask the teacher for help” (parent-version). Participants rate the items on a 6-point Likert scale (1=strongly disagree to 6=strongly agree), and higher scores on performance- or learning-orientation items indicate their preferred orientations. The Cronbach α in previous study were 0.79 and 0.78 for performance-orientation and learning-orientation, respectively [16].

Academic self-efficacy will be measured by a 5-item scale that was adapted from the part of Patterns of Adaptive Learning Survey [51]. Children will rate the extent to which the skills taught in class could be mastered and the work could be done on a 6-point Likert scale (1=strongly disagree to 6=strongly agree). The sample question is “I’m certain I can master the skills taught in class.” A higher score means a higher level of academic self-efficacy.

The motivation for applying the contents learned from the intervention will be assessed right after the intervention. We will ask participants the extent to which they would like to apply the intervention contents in life, and the extent to which they would like to improve their ability to cope with challenges on a 6-point Likert scale from 1=not very likely to 6=very likely.

The intervention feedback scale will be used to assess the acceptability of parent and child interventions. The scale will ask parents and children to indicate how much they enjoyed, understood, and felt helped by the program; whether they would recommend the program to a friend; whether they found the program interesting and fun; whether they agree with the program’s message; whether the program is a burden; and the overall acceptability regarding this program [26]. Participants rate the items on s 5-point Likert scale (1=strongly disagree to 5=strongly agree). The items will be analyzed collectively as a single item.

### Covariates

Sociodemographic information will be collected from parents and will cover a range of participant characteristics (gender, age, grade, and socioeconomic status) at baseline to examine variability between the groups.

### Data Analysis

An intention-to-treat approach will be adopted. A 2-level analysis will be used to account for the cluster randomization [52]. To examine the effects of the interventions, multilevel regression will be used to test the group effect, time effect, and interaction effect on outcome measures. Additionally, we will calculate effect sizes using estimated marginal means; these effect sizes compare mean change scores (Cohen *d*), reflecting the standardized levels of change in each outcome from baseline to 2 follow-ups for youths receiving the mindset versus active control interventions. A 95% CI will be provided for each effect size. SPSS (version 26; IBM Corp) will be used for all statistical analysis.

### Ethical Considerations

Research ethical approval has been obtained from the Hong Kong Polytechnic University institutional review board for Ethical Review (HSEARS20211009001). Participation will be voluntary. Informed written consent will be obtained from participating parents and students. Participants will be reminded that they have the right to terminate the intervention or abstain from responding to any question on the questionnaire without providing a reason, and they will be assured of confidentiality. Additional information for referral services will be provided to participants with clinical-level mental health needs. All identifiable information will be removed, and codes will replace names to ensure privacy. All personally identifiable data will be stored on a secure server and destroyed 3 years after the study ends. Participants’ data will not be identifiable in any publication or reporting. Each participating family in the study will receive a HK $100 (≈US $12) supermarket voucher after the intervention and another one after the 3-month follow-up, making the total compensation for each family HK $200 (≈US $40).

## Results

We have started school invitation and participation recruitment in September 2023. The first school joined the study in May 2024. 75 students from 3 classes were randomly assigned into 3 groups. Students completed baseline surveys and intervention at school, while parents completed baseline assessment and intervention at home. Those have done the baseline receive reminders to complete the baseline assessment or intervention within 1 week after the baseline. Follow-up assessments will be finished by September. Meanwhile, we will send invitations to other schools until we have a sufficient sample size. The effect size will be calculated based on the data of the first school, and the sample size may be adjusted. We will conduct data collection from September 2024 to April 2025 and complete all follow-up surveys by April 2025.

## Discussion

### Expected Findings

This study develops the first a parent-child single-session integrated mindset intervention for Chinese adolescents which simultaneously targets to promote the growth mindset of intelligence, failure, and emotion among adolescents and their parents. This RCT will provide empirical evidence on the effectiveness of the intervention. The hypothesized main finding would be that PC-SMILE intervention has better intervention outcomes, including decreased anxiety and depression symptoms, reduced hopelessness, enhanced academic self-efficacy, psychological well-being, and parent-child interactions and relationships, than child intervention only or a waitlist control group.

As one of the first few attempts on parental mindsets, we will compare the effectiveness of parent-child intervention and child intervention only. This will be the unique evidence to show if intervention targeting parental mindset helps improve children’s mental health outcome.

The challenge of SSIs is that it may be difficult to have sustainable and long-term effects. Thus, we measure the 2-week and 3-month follow-up with the latter as the primary outcome. This evidence-based program is novel, feasible and scalable to meet the needs of a large number of young people across the country. The digital delivery of PC-SMILE ensures that it has the potential to be scalable to provide consistent, evidence-based, and prevention-focused psychoeducation. However, given the scale of the study, a key limitation includes the reliance on self-report measures and lack of objective measurement. Although we measure parent-reported depression and anxiety symptoms and parent-child interaction time, the general mode of measure is self-report. Future directions would be incorporating objective measurements alongside self-report measures, such as behavioral observations in schools.

This study has several potential implications. For theoretical implications, the proposed study will elucidate the effectiveness of changing parents and children’s mindsets on adolescent internalizing problems. In addition, this study will provide evidence of the effectiveness of integrated mindset intervention for both parents and children. From a practical or clinical perspective, by instilling both parents and children with a growth mindset, PC-SMILE can potentially help parents better support children with emotional distress. The PC-SMILE can serve as an add-on component of multisession psychosocial treatment. Finally, this study has social implications that PC-SMILE can potentially facilitate better family support and care, thereby reducing the social burden of mental health care needs. As the first attempt to develop and implement a web-based, single-session, parent-child intervention to promote youth and parental well-being in Hong Kong, this study will provide evidence-based service recommendations on brief interventions for young people and their parents in Hong Kong and beyond.

### Conclusions

The current study uses a 3-arm RCT to examine the effectiveness of PC-SMILE in reducing depression and anxiety symptoms among children in Hong Kong. As an integrated intervention, PC-SMILE is highly likely to be a potent mindset intervention. If proven to be effective, PC-SMILE would be a low-cost, easily accessible self-help tool for thousands of families that could significantly promote youth and parental well-being.

## Appendix

Multimedia Appendix 1 Table of assessment elements.

## Abbreviations

C-SMILE: child single-session intervention on mindsets of intelligence, failure, and emotion
CONSORT: Consolidated Standards of Reporting Trials
PC-SMILE: parent and child single-session intervention on mindsets of intelligence, failure, and emotion
RCT: randomized controlled trial
SSI: single-session intervention

## Data Availability

The data sets produced and analyzed in this study are not publicly accessible because of the confidentiality. However, the deidentified data can be obtained from the corresponding author upon reasonable request.
