# Supplementary material for: Effects of a Parent-Child Single-Session Growth Mindset Intervention on Adolescent Depression and Anxiety Symptoms: Protocol of a 3-Arm Waitlist Randomized Controlled Trial
Source: JMIR Res Protoc. 2024 Aug 30;13:e63220. doi: 10.2196/63220 (PMC11399747; doi:10.2196/63220)
Supplement: Multimedia Appendix 1 [file resprot_v13i1e63220_app1.doc]

| **Assessment** | **Pretest**  (baseline) | **Post-test**  (baseline) | **Post-test 1**  (2-weeks) | **Post-test 2**  (3-months) |
| --- | --- | --- | --- | --- |
| ***Youth Self-report*** |  |  |  |  |
| Mindset about intelligence | ✓ | ✓ | ✓ | ✓ |
| Mindset about failure | ✓ | ✓ | ✓ | ✓ |
| Mindset about emotion | ✓ | ✓ | ✓ | ✓ |
| RCADS-25: child version | ✓ | - | ✓ | ✓ |
| Hopelessness | ✓ | - | ✓ | ✓ |
| Psychological well-being | ✓ | - | ✓ | ✓ |
| Parent-child interactions | ✓ | - | ✓ | ✓ |
| Parent-child relationships | ✓ | - | ✓ | ✓ |
| Perceived parent learning versus performance orientation | ✓ | - | ✓ | ✓ |
| Academic self-efficacy | ✓ | - | ✓ | ✓ |
| Motivation for applying the contents learned from the intervention | - | ✓ | - | - |
| Intervention feedback | - | ✓ | - | - |
| Demographics | ✓ | - | - | - |
| ***Parent Report*** | | | | |
| Mindset about intelligence | ✓ | ✓ | - | ✓ |
| Mindset about failure | ✓ | ✓ | - | ✓ |
| Mindset about emotion | ✓ | ✓ | - | ✓ |
| RCADS-25: parent version | ✓ | - | ✓ | ✓ |
| Parental performance/learning-oriented responses | ✓ | ✓ | - | - |
| Psychological well-being | ✓ | - | ✓ | ✓ |
| Parent-child relationship | ✓ | - | ✓ | ✓ |
| Intervention feedback | - | ✓ | - | - |
| Motivation for applying the contents learned from the intervention | - | ✓ | - | - |
| Demographics | ✓ | - | - | - |
